# Resistance of *Anopheles stephensi* to selected insecticides used for indoor residual spraying and long-lasting insecticidal nets in Ethiopia

**DOI:** 10.1186/s12936-023-04649-5

**Published:** 2023-07-27

**Authors:** Abebe Teshome, Berhanu Erko, Lemu Golassa, Gedeon Yohannes, Seth R. Irish, Sarah Zohdy, Melissa Yoshimizu, Sisay Dugassa

**Affiliations:** 1 National Malaria Elimination Programme, Ministry of Health, Ethiopia, P.O._Box 1234, Addis Ababa, Ethiopia; 2 Aklilu Lemma Institute of Pathobiology, Addis Ababa University, P.O._Box 1176, Addis Ababa, Ethiopia; 3 Department of Zoological Sciences, Addis Ababa University, P.O._Box 1176, Addis Ababa, Ethiopia; 4 Swiss Tropical and Public Health Institute (Swiss TPH), 4123 Allschwil, Switzerland; 5 US President’s Malaria Initiative, Centers for Disease Control and Prevention, Atlanta, GA USA; 6 US President’s Malaria Initiative, US Agency for International Development, Washington, DC USA

**Keywords:** *Anopheles stephensi*, Long-lasting insecticidal nets, Indoor residual spraying, Susceptibility, Malaria, Vector control, Resistance

## Abstract

**Background:**

Malaria, transmitted by the bite of infective female *Anopheles* mosquitoes, remains a global public health problem. The presence of invasive *Anopheles stephensi*, capable of transmitting *Plasmodium vivax* and *Plasmodium falciparum*, was first reported in Ethiopia in 2016. The ecology of this mosquito species differs from that of *Anopheles arabiensis*, the primary malaria vector in Ethiopia. This study aimed to evaluate the efficacy of selected insecticides, which are used in indoor residual spraying (IRS) and selected long-lasting insecticidal nets (LLINs) for malaria vector control against adult *An. stephensi*.

**Methods:**

*Anopheles stephensi* mosquitoes were collected as larvae and pupae from Awash Subah Kilo Town and Haro Adi village, Ethiopia. Adult female *An. stephensi*, reared from larvae and pupae collected from the field, aged 3–5 days were exposed to impregnated papers of IRS insecticides (propoxur 0.1%, bendiocarb 0.1%, pirimiphos-methyl 0.25%), and insecticides used in LLINs (alpha-cypermethrin 0.05%, deltamethrin 0.05% and permethrin 0.75%), using diagnostic doses and WHO test tubes in a bio-secure insectary at Aklilu Lemma Institute of Pathobiology, Addis Ababa University. For each test and control tube, batches of 25 female *An. stephensi* were used to test each insecticide used in IRS. Additionally, cone bioassay tests were conducted to expose *An. stephensi* from the reared population to four brands of LLINs, MAGNet™ (alpha-cypermethrin), PermaNet^®^ 2.0 (deltamethrin), DuraNet^©^ (alpha-cypermethrin) and SafeNet^®^ (alpha-cypermethrin). A batch of ten sugar-fed female mosquitoes aged 2–5 days was exposed to samples taken from five positions/sides of a net. The data from all replicates were pooled and descriptive statistics were used to describe features of the data.

**Results:**

All *An. stephensi* collected from Awash Subah Kilo Town and Haro Adi village (around Metehara) were resistant to all tested insecticides used in both IRS and LLINs. Of the tested LLINs, only MAGNet™ (alpha-cypermethrin active ingredient) caused 100% knockdown and mortality to *An. stephensi* at 60 min and 24 h post exposure, while all other net brands caused mortality below the WHO cut-off points (< 90%). All these nets, except SafeNet^®^, were collected during LLIN distribution for community members through the National Malaria Programme, in December 2020.

**Conclusions:**

*Anopheles stephensi* is resistant to all tested insecticides used in IRS and in the tested LLIN brands did not cause mosquito mortality as expected, except MAGNet. This suggests that control of this invasive vector using existing adult malaria vector control methods will likely be inadequate and that alternative strategies may be necessary.

## Background

Malaria, transmitted by the bite of infective female *Anopheles* mosquito, is a global public health problem that mainly affects tropical countries [1, 2]. Globally, there are over 3530 mosquito species in 43 genera and of these, vectors of human malaria parasites belong to the genus *Anopheles* [3].

In Ethiopia, *Anopheles arabiensis* is the primary malaria vector, while *Anopheles pharoensis*, *Anopheles funestus* and *Anopheles nili* are secondary vectors [4]. The invasive mosquito species, *Anopheles stephensi* was first reported in the country in 2016 and has exhibited the potential of transmitting *Plasmodium falciparum* and *Plasmodium vivax* [1, 6, 7]. *Anopheles stephensi* has also been reported as invasive from other countries including the African countries of Djibouti (2012), the Sudan (2016), Somalia (2019) and Nigeria (2020) [8, 9]. The broad geographic distribution to date has raised concern about appropriate and effective vector control strategies to target this invasive species particularly within the African context [5, 10].

Unlike the native malaria vectors in Africa, *An. stephensi* is adapted to urban and peri-urban settings in man-made habitats such as overhead tanks, ditches, cement tanks (birka) and canals as larval sites [1, 5, 11]. It feeds on both humans and animals, and blood meal data suggests a potential preference for the latter [12], and it may exhibit more outdoor feeding than indoor feeding [1, 6]. More importantly, *An. stephensi* from other areas in Ethiopia has been reported to be resistant to most of the insecticides used for indoor residual spraying (IRS) and long-lasting insecticidal nets (LLINs) [1, 13]. The mosquito samples in this study were collected from different regions than previous studies and used to determine insecticide resistance status of these populations to IRS and LLIN active ingredients and for testing the bio-efficacy of bed net products against the species.

## Methods

### *Anopheles stephensi* larval and pupal collection sites

**Fig. 1:**
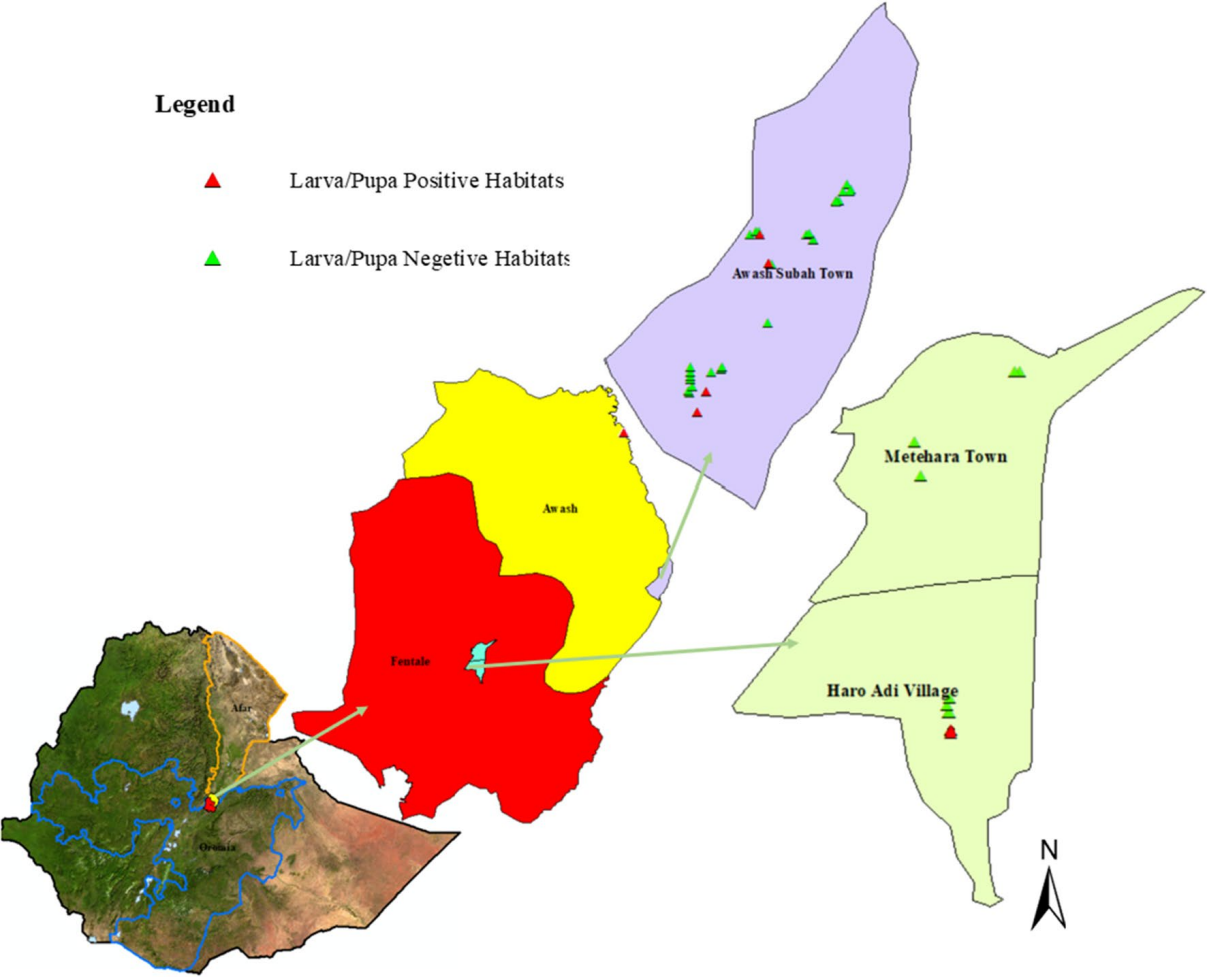
Map of Ethiopia showing the study sites

Larvae and pupae of *An. stephensi* were collected from Awash Subah Kilo Town (also called Awash Sebat Kilo Town) and Haro Adi village around Metehara from January 2021 to June 2021, which is mostly a dry season, except the minor rains in March–April, in these areas. Awash Subah Kilo Town (08°59′24.50″ N, 40°9′54.46″ E) is located in Administrative Zone 3 of the Afar Regional State, just above a gorge of the Awash River, after which it is named. The town lies on the Addis Ababa–Djibouti Railway line at about 217 km from Addis Ababa. This town is the largest settlement in Awash Fentale district, situated at an elevation of 986 m. Haro Adi village is located near Metehara Town. Metehara Town (08°52′35.29″ N, 39°55′8.58″ E) is located in central Ethiopia in the East Shewa Zone of the Oromia Regional State, situated at an elevation of 947 m above sea level. Haro Adi village, from where the larvae and pupae of *An. stephensi* were collected, is a village located approximately two kilometres to the south of Metehara Town along Lake Beseka (Fig. 1).

### Rearing *Anopheles stephensi* mosquitoes

The larvae and pupae collected from the field were transported in plastic containers and reared to adults in the insectary at Aklilu Lemma Institute of Pathobiology, Addis Ababa University (AAU-ALIPB). The insectary has two secured doors, with a double door at the entrance and each separate unit of the insectary has its own door and sealed glass windows, which prevent mosquitoes from escaping. During mosquito rearing temperature and relative humidity of the lab were monitored. In the insectary, larvae were transferred into white plastic trays which were covered with mesh to prevent emerging adults. Larvae were fed by adding dry baker’s yeast (Saf-instant^®^), approximately half a teaspoon at a time, to the tray, and 5 min later, the tray was swirled to distribute the yeast powder and prevent suffocation from undiluted/accumulated powder [14].

Pupae were removed with plastic pipettes and transferred into a beaker with fresh deionized water and then transferred to adult holding cages daily. Adults in the cage were provided with 10% sugar solution using wetted cotton placed on the top of the mesh cage. The cotton was maintained wet so that mosquitoes could feed on the sugar ad libitum. The cotton balls were changed every 5 to 6 days, in order to avoid growth of mold spores and/or fungus [14]. Concurrent with sugar feeding, 3–7 days old female mosquitoes were fed on live rabbit blood meals twice per week (ethical approval was obtained from AAU-ALIPB Ethical Review Board). Water-filled petri dishes and/or wet filter papers supported with cotton and placed on petri dishes were provided for mosquitoes to lay eggs on. The bioassay tests were conducted up to the seventh generation, with intention to have susceptible colony to be tested against LLINs products. But, for the interest of time, the bioassay was conducted on the eighth and later generation of mosquitoes where confirmed susceptibility is not attained.

Based on WHO guideline, if the 24-h mortality in tested mosquitoes is ≥ 98%, it is considered susceptible; however, if it is from 90 to 97% and < 90%, it is considered as possible and confirmed resistance, respectively.

### *Anopheles stephensi* species identification

Morphological identification of mosquitoes was done using dissecting microscope. Before commencing any bioassay tests 30 adult female mosquitoes were randomly aspirated from cages and transferred into paper cup. Then these mosquitoes exposed to and killed by chloroform (LABORT^®^). Dead mosquitoes were transferred to petri dish and placed under a stereomicroscope at 40× and identified using identification key [15]. The identification was repeated on all exposed mosquitoes after reading the result. The specimens were neither stored nor sequenced for further molecular confirmation because of resource limitations.

### Efficacy of long-lasting insecticide treated net products against adult stage of *Anopheles stephensi*

The LLINs for test were obtained both from Aklilu Lemma Institute of Pathobiology (ALIPB) and Amibara district health office in Afar Regional State. Four net products, MAGNet™, PermaNet^®^ 2.0, DuraNet^©^ and SafeNet^®^, were used for the test. The first three were obtained from those imported for distribution through the national programme in 2020. These LLINs were collected from the distribution point prior to distribution to the community. SafeNet^®^, was obtained from ALIPB from the LLIN products collected for efficacy testing purposes. The manufacturing locations of the nets were Indian, Vietnam and China, respectively. DuraNet^©^ product release and expiry dates were August 2020 and August 2023, respectively. MAGNet™ product release and expiry dates were March 2020 and February 2023, respectively.

MAGNet™ was impregnated with alpha-cypermethrin of 5.8 g/kg (261 mg/m^2^) and DuraNet^©^ was impregnated with alpha-cypermethrin of 5.8 g/kg. SafeNet^®^ is also impregnated with alpha-cypermethrin of 5.0 g/kg (200 mg/m^2^). PermaNet^®^ 2.0 was impregnated with deltamethrin of 1.4 g/kg (56 mg/m^2^). Due to the SafeNet^®^ and PermaNet^®^ 2.0 product tags not including insecticide information, details on insecticide impregnation were obtained via WHO PQ/manufacturer websites. The tags on all net products did not include information of denier.

Five samples, one from each net panel (upper, head, feet, right and left), were taken with the tag referenced as the head position. The size of each sample taken from each net panel was 25 cm by 25 cm. Two WHO bioassay cones were used for each of the net samples taken from the aforementioned positions. Five sugar-fed 2–5 day old female adult *An. stephensi* were placed in each cone, resulting in ten mosquitoes tested per net sample, and were exposed for three minutes [16]. Simultaneously, as a negative control, two groups of ten sugar-fed 2–5 day old female adult *An. stephensi* were exposed via WHO cone bioassay to an untreated net sample (a net not treated with any insecticide). After the three minutes exposure time, mosquitoes were transferred to holding cups and were immediately provided with 10% sugar solution. Knockdown and mortality were recorded at 60 min and 24 h post exposure. The test was repeated, simultaneously with control groups, on two other nets of the same product on different days, resulting in a total of three nets tested for each net product. The investigation was done, in June 2021, on unused and unwashed nets and conducted under controlled laboratory conditions.

### Susceptibility test of *Anopheles stephensi*

Six insecticide-impregnated papers (propoxur 0.1%, bendiocarb 0.1%, pirimiphos-methyl 0.25%, deltamethrin 0.05%, alpha-cypermethrin 0.05% and permethrin 0.75%), all with impregnation date of February 2020 and date of expiry in February 2023, were obtained from Ethiopian Public Health Institute (EPHI).

The study was conducted as per the World Health Organization (WHO) standard procedures for conducting susceptibility test [17]. Six tubes for holding (labeled green), four tubes for treatment (labelled red) and two control tubes (labelled yellow) were set up prior to testing. Batches of 25 (3–5 day old non-blood fed) reared from F0, F1, F2… female mosquitoes were taken from cages using mouth aspirators and transferred into the tubes. At the end of 1 h resting period, they were transferred and exposed for 1 h [17]. For the pyrethroids insecticides, mosquitoes unable to stand or move in a coordinated manner, or unable to fly were recorded as knocked down. After 1 h, mosquitoes were transferred back into holding tubes with untreated papers and 10% sugar water. During the 24-h post-exposure holding period, provided with 10% sugar water, tubes were kept in a cardboard shelter in the laboratory with maintained temperature and relative humidity. Insecticide resistance is the ability of insects to survive exposure to a standard dose of insecticide, owing to physiological or behavioural adaptation [17].

### Data analysis

Data were recorded using the WHO susceptibility test result recording form. Data from all replicates were pooled and entered into an Excel spreadsheet for analysis using STATA version 14.0. Logistic regression for mortality or survival of mosquitoes as an effect of exposure to insecticides and odds ratio was calculated for insecticides and by sites of mosquito origin (Table 3).

### Data quality assurance

Data quality was maintained by strictly implementing the control of other factors, such as temperature, humidity and conducting the test as per the laboratory procedures. In addition, data were rechecked for proper capturing at recording, organizing, cleaning and analysis steps.

### Ethical considerations

This study involved no human study participants, and it was implemented after obtaining ethical clearance (Ref. No. ALIPB IRB/40/2013/21) from the IRB of Aklilu Lemma Institute of Pathobiology, Addis Ababa University.

This study aimed to determine the efficacy of insecticides used in IRS and selected LLINs products against adult *An. stephensi*. All mosquitoes collected from Awash Subah Kilo Town and Haro Adi village (Fig. 1) and tested were confirmed to be *An. stephensi* by morphological identification methods.

## Results

### Efficacy of insecticides against adult *Anopheles stephensi*

The knockdown and mortality effect induced by insecticides to exposed mosquitoes is presented in Table 1. In Awash Subah Kilo Town, 236 (39.3%) out of the 600 exposed mosquitoes were knocked down within 1 h of exposure. In Haro Adi village, from a total of 600 mosquitoes exposed to pyrethroids, 271 (45.2%) were knocked down within 1 h of exposure.

**Table 1:** Observed knockdown and mortality effect by insecticides on adult *Anopheles stephensi* reared from Awash Subah Kilo Town and Haro Adi village and susceptibility status as compared to WHO criteria, June 2021

Site	Insecticide diagnostic concentration (%)	No. exposed adult *An. stephensi*	Knockdown at 1 h, n (%)	No. (%) mortality at 24 h^c^	WHO criteria
Awash Subah Kilo Town	^a^Alpha-cypermethrin 0.05%	100	60	21 (21%)	Confirmed resistance
	^a^Deltamethrin 0.05%	100	88	84 (84%)	Confirmed resistance
	^a^Permethrin 0.75%	100	88	64 (64%)	Confirmed resistance
	^b^Bendiocarb 0.1%	100	0	3 (3%)	Confirmed resistance
	^b^Propoxur 0.1%	100	0	8 (8%)	Confirmed resistance
	^b^Pirimiphos-methyl 0.25%	100	0	None died	Confirmed resistance
Total		600	236 (39.3%)		
Haro Adi village	^a^Alpha-cypermethrin 0.05%	100	71	77 (77%)	Confirmed resistance
	^a^Deltamethrin 0.05%	100	95	81 (81%)	Confirmed resistance
	^a^Permethrin 0.75%	100	94	74 (74%)	Confirmed resistance
	^b^Bendiocarb 0.1%	100	0	1 (1%)	Confirmed resistance
	^b^Propoxur 0.1%	100	8	9 (9%)	Confirmed resistance
	^b^Pirimiphos-methyl 0.25%	100	3	5 (5%)	Confirmed resistance
Total		600	271 (45.2%)		

^a^Insecticides used in LLIN impregnation

^b^Insecticides used in indoor residual spraying

^c^Confirmed resistance when mortality at 24 h < 90%

All *An. stephensi* mosquitoes reared from larvae and pupae collected from both Awash Subah Kilo Town and Haro Adi village were resistant to all tested insecticides (Table 1). The mortality against all insecticides used for IRS, bendiocarb (0.1%), propoxur (0.1%) and pirimiphos-methyl (0.25%), the mortalities was under 10%. This species is also resistant to the three pyrethroids insecticides tested with all mortalities falling below 90% WHO threshold for confirmed resistance. From the total of 600 mosquitoes used as controls throughout the bioassay, only 10 (1.7%) mosquitoes’ died within 24 h, therefore, no corrections were needed. The induced mortality, as sorted by insecticide, to exposed mosquitoes has a statistically significant difference (P < 0.0001) (Table 2).

**Table 2:** Statistical difference, by insecticide, on Adult *An. stephensi* mosquito mortality caused by insecticides collected as larvae and pupae from Awash Subah Kilo Town and Haro Adi village combined, June 2021

Insecticide	No. exposed mosquito	No.(%) mortality^a^	Chi-square	*P*-value
Alpha-cypermethrin 0.05%	200	98 (23%)	563.112	< 0.0001
Bendiocarb 0.1%	200	4 (0.9%)		
Deltamethrin 0.05%	200	165 (38.6%)		
Permethrin 0.75%	200	138 (32.3%)		
Pirimiphos-methyl 0.25%	200	5 (1.2%)		
Propoxur 0.1%	200	17 (4%)		

^a^% mortality attributed by a single insecticide is calculated from the total mortality induced by all insecticides within 24-h post exposure

Logistic regression was used to assess differences in mortality between insecticides and sites of origin as a factor at 95% confidence interval. The odds of mortality from insecticides were calculated by taking alpha-cypermethrin 0.05% insecticide and Awash Subah Kilo Town as referent for comparing with their counterparts. The odds ratio of mosquitoes to die within 24 h post exposure as a result of exposure to deltamethrin 0.05% was five times higher than those exposed to alpha-cypermethrin 0.05%, or those mosquitoes exposed to alpha-cypermethrin had 96% chance of survival as compared to those exposed to deltamethrin 0.05%. Those mosquitoes exposed to bendiocarb and pirimiphos-methyl had 98% and 96.7% chance of survival, respectively, as compared to those exposed to deltamethrin. The odds ratio of mortality for mosquitoes from Haro Adi village was three times higher than those from Awash Subah Kilo Town, P < 0.0001 (OR = 2.58; 95% CI 1.836–3.63) (Table 3).

**Table 3:** Odds ratio of insecticide induced mortality within 24 h post exposure to adult *An. stephensi* mosquitoes reared from larvae and pupae collected from Awash Subah Kilo Town and Haro Adi village, June 2021

Insecticide	Odds ratio^a^	*p*-value	95% confidence interval
Bendiocarb 0.1%	0.02	< 0.0001	0.0068165–0.05395
Deltamethrin 0.05%	5.29	< 0.0001	3.3040–8.4645
Permethrin 0.75%	2.42	< 0.0001	1.5928–3.689796
Pirimiphos-methyl 0.25%	0.02	< 0.0001	0.00945–0.06156
Propoxur 0.1%	0.09	< 0.0001	0.049534–0.1579
Haro Adi village site	2.58	< 0.0001	1.8356–3.627772

^a^Alpha-cypermethrin 0.05% and Awash Subah Kilo Town are taken as referent to compare with respective counterparts

Mosquitoes from Haro Adi village had a 2.581 times greater chance of mortality than those from Awash Subah Kilo Town as a result of exposure to these insecticides. Mosquitoes exposed to deltamethrin 0.05% had a 5.29 times greater chance of mortality than mosquitoes exposed to alpha-cypermethrin 0.05%.

### Efficacy of long-lasting insecticide treated net products against adult *Anopheles stephensi*

There was a statistically significant difference (p < 0.001) between LLINs brands in inducing knockdown and mortality of adult *An. stephensi* mosquitoes (Table 4). Exposure to MAGNet™ samples resulted in 100% knockdown and mortality at 1 and 24 h post exposure. Exposure to DuraNet^©^ samples resulted in 84.7% knockdown and 80.7% mortality; and exposure to PermaNet^®^ 2.0 samples resulted in 74.0% knockdown and 80.0% mortality. The induced knockdown and mortality by SafeNet^®^ was 24.0% and 24.7%, respectively. From the control group 0.42% (n = 1) mosquito mortality was observed within 24 h, therefore, no correction was necessary.

**Table 4:** Bio-efficacy of long-lasting insecticide treated net products against adult *Anopheles stephensi* reared from larvae and pupae collected from Awash Subah Kilo Town and Haro Adi village, June 2021

Brands of Net	Insecticide	Amount of insecticide (manufacturer-given )	No. of exposed mosquito	Knockdown at 1 h, n (%)	χ^2^	P-value	Mortality at 24 h, (%)	χ^2^	P-value
SafeNet^®^	Alpha-cypermethrin	5.0 g/kg (200 mg/m^2^)	150	36 (24.0%)	234.8	< 0.001	37 (24.7%)	231.9	< 0.001
MAGNet™	Alpha-cypermethrin	5.8 g/kg (261 mg/m^2^)	150	150 (100.0%)			150 (100.0%)		
DuraNet^©^	Alpha-cypermethrin	5.8 g/kg	150	127 (84.7%)			121 (80.7%)		
PermaNet^®^ 2.0	Deltamethrin	1.4 g/kg (56 mg/m^2^)	150	111 (74.0%)			120 (80.0%)		

## Discussion

This study aimed to evaluate the efficacy of selected insecticides used for IRS and LLINs against adults of the invasive *An. stephensi* collected in Ethiopia.

In this study, all *An. stephensi* mosquitoes reared from larvae and pupae collected from Awash Subah Kilo Town and Haro Adi village were resistant to all six insecticides tested. This is in agreement with previous reports of resistance in *An. stephensi* from other areas (and all other local malaria vectors) in Ethiopia [18, 19]. In Kebridehar, Somali Regional State, resistance to bendiocarb, propoxur, pirimiphos-methyl, multiple pyrethroids, as well as DDT and malathion, have been reported [13, 20]. Findings from these two studies from various locations in Ethiopia have shown confirmed resistance to most of the insecticides used to target adult mosquitoes.

In contrast, insecticide resistance monitoring conducted in various locations in India, from 2004 to 2007, showed that adult *An. stephensi* were susceptible to deltamethrin, and exhibited variable levels of resistance to DDT and malathion [21]. Furthermore, testing from India indicates the level of resistance is at least moderate. *Anopheles stephensi* resistance to pyrethroids, organochlorines, carbamates, and organophosphates has also been reported from Afghanistan, Iran, India and Pakistan [22]. *Anopheles stephensi* have developed both target site and metabolic resistance mechanisms [22] to various insecticides.

In this study, MAGNet™ was the only net product which caused 100% mortality of adult *An. stephensi* mosquito within 24 h, post exposure. Despite being treated with the same active ingredient (alpha-cypermethrin) as MAGNet™, both DuraNet^©^ and SafeNet^®^ resulted in knockdowns below the cut-off point (≥ 95%) and 24 h post exposure mortality (80.0% and 24.7%, respectively) which were significantly less than MAGNet™ and below the efficiency cut-off point (≥ 80% mortality). PermaNet^®^ 2.0, treated with deltamethrin, exhibited both knockdown and mortality below the respective WHO cut-off points [16, 23]. The results of PermaNet^®^ 2.0 from this study were not in agreement with a study conducted against *Anopheles culicifacies* and *An. stephensi* in India, in which mortality of mosquitoes of both species remained > 80% to Olyset Net and PermaNet 2.0 even after use and up to 20 hand washings [24]. The difference in results could be explained in actual insecticide impregnation dosage differences, quality of the insecticide, netting material used, and/or because of transportation and storage and handling variables. These differences are also likely due to age variance and resistance status of the test mosquitoes. The result of this laboratory-based study as compared to the nets tested for efficacy in India show that many variables impact efficacy as measured by knockdown and mortality via bioassay. In the India study [24], it was observed that the efficacy of insecticide impregnated in the LLINs diminishes faster when the net receives washing, particularly machine washings. However, given the efficacy for all net products except MAGNet™, in this study were below WHO cut-offs using brand new nets, it is unclear what the efficacy impact of washing to those net products might be.

The invasion of *An. stephensi* into new geographic regions [2, 5, 6, 22] and the observed distribution of resistance to insecticides [22] is reaffirmed by data from this study. The findings from *An. stephensi* resistance studies thus far, collectively confirm the species is resistant to many of the insecticides used to control adult mosquitoes across vast geographic areas. As such, existing malaria adult vector control methods significantly less effective and alternative control methods, such as larval source management [1, 25] will be needed to manage this invasive species. The limitations of the present study were reliance on morphological identification of mosquitoes, and further molecular analysis was not conducted due to resource limitations.

## Data Availability

All datasets on which the conclusions of this study relied on are presented in this paper.

## Abbreviations

AAU: Addis Ababa University
ALIPB: Aklilu Lemma institute of pathobiology
EPHI: Ethiopian public health institute
GPS: Global positioning system
IRB: Institutional review board
IRS: Indoor residual spraying
LLINs: Long lasting insecticide impregnated Nets
RH: Relative humidity
WHO: World Health Organization
